# Effect of intra-articular corticosteroid injections for osteoarthritis on the subsequent use of pain medications: a UK CPRD cohort study

**DOI:** 10.1093/rheumatology/keaf126

**Published:** 2025-03-01

**Authors:** Samuel Hawley, Albert Prats-Uribe, Gulraj S Matharu, Antonella Delmestri, Daniel Prieto-Alhambra, Andrew Judge, Michael R Whitehouse

**Affiliations:** Musculoskeletal Research Unit, Translational Health Sciences, Bristol Medical School, University of Bristol, Learning & Research Building Level 1, Bristol, UK; Centre for Statistics in Medicine, Nuffield Department of Orthopaedics, Rheumatology and Musculoskeletal Sciences, Botnar Research Centre, Oxford, UK; Musculoskeletal Research Unit, Translational Health Sciences, Bristol Medical School, University of Bristol, Learning & Research Building Level 1, Bristol, UK; Centre for Statistics in Medicine, Nuffield Department of Orthopaedics, Rheumatology and Musculoskeletal Sciences, Botnar Research Centre, Oxford, UK; Centre for Statistics in Medicine, Nuffield Department of Orthopaedics, Rheumatology and Musculoskeletal Sciences, Botnar Research Centre, Oxford, UK; Musculoskeletal Research Unit, Translational Health Sciences, Bristol Medical School, University of Bristol, Learning & Research Building Level 1, Bristol, UK; Centre for Statistics in Medicine, Nuffield Department of Orthopaedics, Rheumatology and Musculoskeletal Sciences, Botnar Research Centre, Oxford, UK; National Institute for Health Research (NIHR), Bristol Biomedical Research Centre, University Hospitals Bristol and Weston NHS Foundation Trust and University of Bristol, Bristol, UK; Musculoskeletal Research Unit, Translational Health Sciences, Bristol Medical School, University of Bristol, Learning & Research Building Level 1, Bristol, UK; National Institute for Health Research (NIHR), Bristol Biomedical Research Centre, University Hospitals Bristol and Weston NHS Foundation Trust and University of Bristol, Bristol, UK

**Keywords:** osteoarthritis, steroid injection, prescribing, pain management, pharmaco-epidemiology

## Abstract

**Objectives:**

To estimate the effect of intra-articular corticosteroid injection (IACI) for osteoarthritis on longer-term incidence of pain medications.

**Methods:**

We conducted a cohort study of patients registered in the UK Clinical Practice Research Datalink (CPRD) GOLD primary care database with an incident diagnosis of knee, hip, hand or shoulder osteoarthritis between 2005 and 2019. Exposure of interest was single or repeated use of IACI (analysed separately). Main outcome measures were five-year incidence of uncombined opioids, opioid-nonopioid analgesic combinations, oral corticosteroids, paracetamol, oral non-steroidal anti-inflammatory drugs (NSAIDs) and topical NSAIDs. Instrumental variable (IV) analysis was used, given this methodology can account for strong and unmeasured confounding. Secondary analyses used propensity-score matching and Cox regression.

**Results:**

Amongst 74 527 knee osteoarthritis patients, IACI use was associated with lower subsequent prescribing of most pain medications studied, including opioid-nonopioid analgesic combinations following single IACI [number needed to treat (NNT) = 5 (95% CI: 5–6), *P* < 0.001] and uncombined opioids following repeat IACI [NNT = 12 (8–546), *P* = 0.049]. Amongst 15 092 hand osteoarthritis patients, single IACI was associated with reduced use of opioid-nonopioid combinations, paracetamol and oral NSAIDs. Secondary analyses confirmed lower incidence rates of opioid-nonopioid combinations after single IACI for knee [hazard ratio (HR) = 0.88 (0.81–0.96)], hip [HR = 0.76 (0.62–0.92)], hand [HR = 0.77 (0.61–0.98)] or shoulder [HR = 0.72 (0.53–0.99)] osteoarthritis.

**Conclusions:**

IACI for knee or hand osteoarthritis showed lower incidence of several pain medications over the longer-term relative to no IACI use. Secondary findings suggest IACI may be effective in reducing longer-term use of opioid-nonopioid analgesic combinations for patients with knee, hip, hand or shoulder osteoarthritis.

Rheumatology key messages

- Intra-articular steroid injections are recommended for osteoarthritis, although more data at non-knee joints is needed.
- In main analyses, use of several pain medications was lower over the longer term following knee or hand steroid injection.
- Steroid injections for knee, hip, hand or shoulder osteoarthritis may reduce longer-term opioid-nonopioid analgesic prescribing.

## Introduction

Osteoarthritis is a common and progressive musculoskeletal condition associated with pain, morbidity, functional decline and reduced quality of life [1–3]. Approximately 10% of adults are estimated to have some degree of joint osteoarthritis [4]. It is a significant public health problem [5] and is associated with substantial healthcare system and societal costs [6]. Due to population ageing and increases in risk factors like obesity, the prevalence of osteoarthritis is increasing [7].

Common treatments for osteoarthritis aim to reduce pain and improve function [8, 9]. Intra-articular corticosteroid injection (IACI) has been a recognized treatment option for over 50 years [9]. Cochrane reports ‘small to moderate’ benefits of IACI for knee osteoarthritis on pain reduction lasting up to six weeks in clinical trials [10], although concludes it is unclear if clinically important benefits last beyond six weeks, and no evidence of an effect at six months [10]. NICE guidance recommends use of IACI for short-term pain relief when other treatments are ineffective or unsuitable, but recognizes there is no evidence of therapeutic benefit lasting beyond three months [11].

However, high heterogeneity of previous findings on IACI effectiveness and low overall quality of evidence has been noted [10–12]. There is a need for better data on longer-term outcomes following IACI [13, 14]. Despite the widespread use of IACI worldwide, most evidence to date pertains to knee osteoarthritis, and future research on the effects of IACI at non-knee joints has been explicitly recommended [11, 15]. Furthermore, whilst OARSI guidelines suggest up to four injections per year could be used, it is acknowledged that data are limited on the appropriate frequency of IACI administration [9].

Our aim was therefore to use quasi-experimental methods applied to routinely collected medical record data to assess the effects of single or repeated use of IACI on longer-term use of pain medications amongst people with knee, hip, hand or shoulder osteoarthritis.

## Methods

### Data and participants

We used primary care health data from the UK Clinical Practice Research Datalink (CPRD) GOLD [16] for the period 1 January 2005 to 28 August 2020. CPRD GOLD contains electronic primary care health records capturing data on patient demographics, symptoms, referrals, test results, diagnoses, clinical measurements and prescribed medicines. At the time of data extraction (September 2020), CPRD GOLD covered 4.8% of the UK population, and cumulatively contained data covering ∼19 million people from over 900 GP practices spread across the country. All-cause mortality data were linked from the Office for National Statistics (ONS) [17] and Index of Multiple Deprivation (IMD) based on Lower Layer Super Output Area of patient’s postcode, and Curator software [18] was used to perform pre-analytical data curation.

Incident osteoarthritis patients were identified using Read code lists and separate cohorts were created for patients with knee, hip, hand or shoulder osteoarthritis. Only patients whose data quality was flagged as acceptable for clinical research and registered at a GP practice with at least one year of ‘up to standard’ (date of which is defined by CPRD in the dataset) clinical records were included [16]. People with osteoarthritis in multiple anatomical joints were excluded (however, bilateral joints of the same type were not excluded) as it was otherwise difficult to confidently ascertain which joint received an IACI from the Read code data. Further exclusion criteria applicable at baseline were: age <20 years old, body mass index (BMI) <15 kg/m^2^, prior referral to orthopedic surgery and osteoarthritis diagnosis after 31 December 2019.

### Exposure

For each joint cohort (knee, hip, hand and shoulder), the exposure of interest was IACI. This was defined using a Read code for IACI in the joint of interest or of unspecified joint location (given the preponderance of such codes and the exclusion of patients with osteoarthritis affecting other sites).

### Outcomes

Primary outcomes were the 5-year incidence of the following pain medication prescriptions: opioids (uncombined prescriptions analysed separately to fixed-dose opioid-nonopioid analgesic combinations [19]), oral corticosteroids, paracetamol, oral non-steroidal anti-inflammatory drugs (NSAIDs) and topical NSAIDs. These were identified using prodcode lists within the CPRD GOLD database.

### Covariates

Covariates were measured using the most recent information available in CPRD GOLD prior to date of osteoarthritis diagnosis, using a look-back period of one year. Demographics and clinical characteristics included age, sex, IMD quintile [20], BMI (<18.5, 18.5–24.9, 25.0–29.9, 30.0–34.9, 35.0–39.9 and ≥40.0 kg/m^2^), smoking status (current, ex-, never) and alcoholic drinking status (current, ex-, never). Prior diagnoses of the following medical conditions were also included: asthma, cancer, chronic obstructive pulmonary disease (COPD), deep vein thrombosis, fracture, lower respiratory tract infection, myocardial infarction, pulmonary embolism, upper respiratory tract infection, urinary tract infection, anaemia, inflammatory arthritis, stroke, diabetes, epilepsy, ischemic heart disease, malabsorption, hyperlipidaemia, hypertension, osteoporosis, chronic renal failure and acute renal failure. Charlson comorbidity score (0, 1, 2 or ≥3) was calculated as previously described [21]. Prior medication use was collected for antiparkinsonian medicines, antiarrhythmics, antibiotics, antidepressants, anticonvulsants, anxiolytics, bisphosphonates, calcium/vitamin D, oral corticosteroids, oral NSAIDs, topical NSAIDs, separate/loose opioids, opioid-nonopioid combinations, paracetamol, proton pump inhibitors, prednisolone, statins, hormone replacement therapy and diuretics. Prior referral to physiotherapy was similarly extracted.

### Statistical analysis

**Figure 1.**
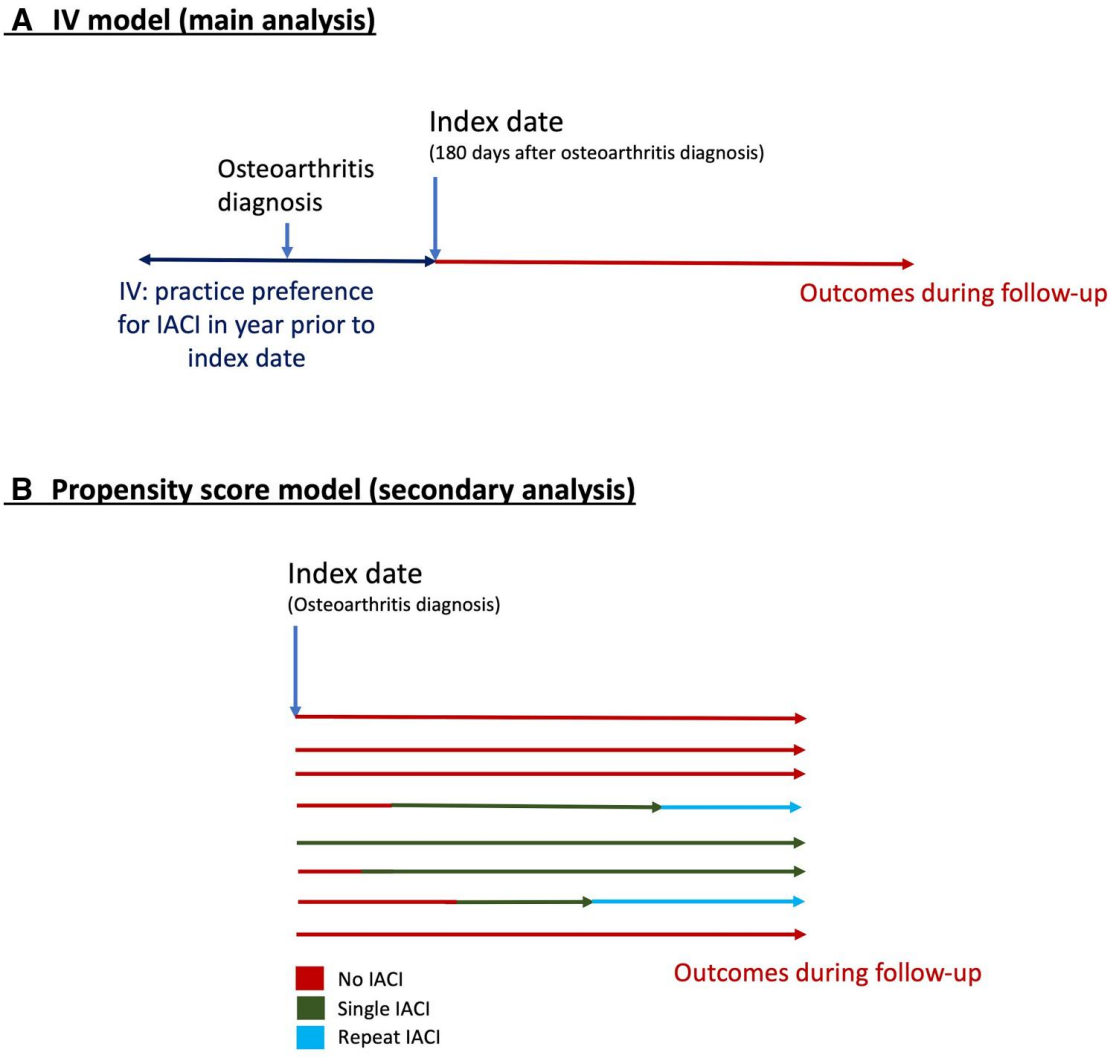
Diagram of model set-up

Instrumental variable (IV) analysis was *a priori* chosen as the primary statistical approach given the suspected strong confounding by indication in an investigation of the ‘real world’ effect of IACI on subsequent use of other pain medication. Important unmeasured confounders not available in the CPRD dataset would also otherwise be unaccounted for, such as pain and radiographic severity, rate of disease progression, physical function, responsiveness to other treatments and quality of life. The IV approach can yield unbiased estimates of treatment effect even in the presence of strong or unmeasured confounding when key assumptions are met [22, 23]. The IV derived and used was a binary indicator of GP practice preference for IACI use during a 1-year exposure window around the date of osteoarthritis diagnosis (Fig. 1). Within this exposure window, GP practice preference was defined as IACI use over the previous 20 incident diagnoses of osteoarthritis at the same joint being greater than the national median use [24]. A shorter look-back of 15 and 10 patients was used for hand and shoulder cohorts, respectively, given the smaller sample sizes for those joints. Preference for single or repeated IACI use was considered separately, compared with preference for non-use. The method was only used where the IV was found to sufficiently predict treatment (odds ratio ≥2 and F-statistic >10 [22, 24]) and was independent of measured potential confounders and missingness [standardized mean difference (SMD) ≤0.1 [25, 26]]. Patients were followed up from model index date (Fig. 1) until the earliest of the following: outcome date, death date, transfer out of the practice, end of five years follow-up or study end. Two-step Poisson regression models were used to produce scaled incidence rate ratios (IRR) of outcomes for IACI use *vs* non-use, alongside 95% CI [24, 27]. We also expressed statistically significant IRRs as the number needed to treat (NNT) with IACI to prevent the occurrence of one new outcome event over five years follow-up, were the association causal [28].

In secondary analyses, the propensity score (i.e. individual conditional probability) of IACI use during follow-up was estimated for each patient using only the covariates available in the dataset [29]. Patients who received IACI during follow-up were matched to similar patients who did not receive IACI, using 1:2 greedy matching within a caliper width of 0.2 standard deviations (SD) of the propensity score [30]. All covariates listed above (i.e. over 50 variables) were included in the logistic regression propensity score estimation. Multiple imputation using chained equations (MICE) was used to impute missing data on BMI, smoking and drinking status [31, 32]. A time-varying exposure approach was taken where follow-up was split according to time-specific exposure status: no IACI, single IACI or repeated IACI. Incidence rates of outcomes with 95% CI were calculated within each exposure period. Association between IACI use and outcomes was estimated using Cox proportional hazards survival analysis, yielding hazard ratios (HRs), using calendar time as the follow-up axis [33]. Results across imputed datasets were pooled using Rubin’s rules [32].

### Sensitivity analyses

Aspects of IV models were altered in sensitivity analyses: (i) using a later index date of one year after osteoarthritis diagnosis date (while still using a 1-year look-back exposure window from index date); (ii) adjustment for geographic region and calendar year of osteoarthritis diagnosis; (iii) restricted to patients aged 60 years and over, given these individuals are eligible to receive free prescriptions on the National Health Service (NHS); (iv) using a secondary outcome measure of repeat (≥10) pain prescriptions during follow-up; and (v) defining exposure to IACI using only joint-specific Read codes.

### Ethics

The study protocol was approved via CPRD Research Data Governance (RDG) process, with number 20_067 and feedback from the Independent Scientific Advisory Committee (ISAC) provided on 28 May 2020. No additional ethical approval was required as this study used pseudo-anonymized routinely collected data.

### Patient and public involvement

We discussed the study proposal and interim findings with members of the Musculoskeletal Research Unit (University of Bristol) Patient Experience Partnership in Research (PEP-R) group: an established forum of eight service users with a range of musculoskeletal conditions, including osteoarthritis.

### Data sharing

Applications to access CPRD GOLD and linked data must be made directly to CPRD in accordance with CPRD’s RDG process.

## Results

### Sample size

Of 163 241 eligible patients identified having osteoarthritis, the total number of patients across all joints used in main IV analyses were 119 580 (73.3%) and 115 532 (70.8%) for single and repeated IACI, respectively (Supplementary Table S1, available at *Rheumatology* online). Sample sizes in secondary propensity score matched analyses are reported in Supplementary Table S2, available at *Rheumatology* online.

### IV diagnostics and patient characteristics

The IV strongly predicted receipt of IACI treatment in all joints. The odds ratios (ORs) and F-values indicating association between the IV and receipt of single IACI in each cohort were: knee (OR 5.51, *F *=* *1177.0); hip (OR 2.69, *F *=* *86.6); hand (OR 3.84, *F *=* *67.7); and shoulder (OR 3.93, *F *=* *16.3). For illustration, in knee osteoarthritis patients, 2748 (9.0%) received IACI amongst those whose GP practice preferred IACI, whilst this number was just 777 (1.8%) for those whose GP practice preferred not to use IACI.

Exploratory analyses indicated there were considerable differences in patient characteristics between those who received an IACI and those who did not, although these were substantially attenuated when comparing characteristics between IV groups (Table 1). Whilst there was sufficient balance (SMD ≤0.1) in patient covariates across IV groups for single IACI in both knee and hand osteoarthritis cohorts, there were some differences observed in the hip and shoulder cohorts, particularly in socio-economic deprivation levels (Table 1). Similar comparisons across IV groups for repeated IACI use indicated acceptable covariate balance only in the knee cohort (results not shown). Given this, subsequent IV analyses were only performed in the knee (single and repeated IACI) and hand (single IACI) osteoarthritis cohorts. In secondary propensity score analyses, acceptable balance (SMD ≤0.1) was found across all covariates between matched IACI users and non-users.

**Table 1.** Selected characteristics of osteoarthritis patients included in main IV analyses of single IACI

Variable	Knee (N = 74 527)^a^		Hip (N = 28 558)^a^		Hand (N = 15 092)^a^		Shoulder (N = 1403)^a^	
	%	SMD^b^	%	SMD^b^	%	SMD^b^	%	SMD^b^
Age in years: mean (S.D.)	65.5 (12.5)	−0.02	66.5 (12.2)	0.00	63.2 (11.2)	0.00	65.6 (13.4)	−0.13
Female	55.9%	−0.01	57.6%	−0.01	71.5%	0.00	48.6%	0.08
Least deprived	9.9%	0.04	9.7%	0.11	14.8%	0.10	5.9%	0.11
Less deprived	9.2%	−0.05	7.9%	0.07	10.3%	−0.09	9.6%	−0.06
Mid deprived	9.6%	−0.05	7.4%	0.08	9.4%	−0.01	10.6%	−0.14
More deprived	8.1%	−0.05	5.8%	0.03	5.8%	−0.07	7.7%	−0.23
Most deprived	6.5%	0.03	4.2%	0.05	4.1%	−0.04	6.3%	−0.10
Deprivation missing	56.7%	0.04	65.1%	−0.19	55.5%	0.03	59.8%	0.24
BMI: <20 kg/m^2^	1.3%	0.01	2.5%	0.03	3.7%	0.02	2.6%	−0.01
BMI 20 < 25 kg/m^2^	15.3%	0.02	21.8%	0.02	28.6%	0.04	21.5%	0.02
BMI 25 < 30 kg/m^2^	31.8%	0.02	34.3%	0.03	33.4%	−0.01	37.2%	0.12
BMI 30 < 35 kg/m^2^	22.5%	−0.01	19.1%	0.01	15.5%	−0.01	19.7%	−0.14
BMI 35 < 40 kg/m^2^	10.3%	0.01	7.0%	0.00	5.5%	0.00	7.0%	−0.01
BMI ≥40 kg/m^2^	6.6%	0.01	3.3%	−0.01	2.2%	−0.02	2.6%	−0.01
BMI missing	12.1%	−0.06	11.9%	−0.08	10.9%	−0.04	9.4%	0.00
Current drinker	60.5%	0.05	63.9%	0.08	64.4%	0.02	61.9%	0.06
Current smoker	11.9%	0.01	13.9%	−0.03	12.5%	−0.05	15.6%	−0.02
Charlson =1	4.5%	0.00	4.6%	0.02	4.1%	0.05	6.2%	−0.05
Charlson =2	3.7%	0.00	3.5%	0.01	2.9%	0.00	3.6%	0.05
Charlson ≥3	1.2%	−0.01	1.1%	0.01	0.8%	−0.03	3.2%	−0.08
Cancer	2.0%	0.01	2.2%	0.00	1.8%	−0.01	2.9%	0.00
Cerebrovascular disease	0.9%	0.01	1.1%	0.01	0.6%	0.00	0.9%	0.04
Chronic obstructive pulmonary disease	3.3%	−0.01	4.3%	−0.02	3.4%	−0.03	5.2%	−0.05
Diabetes	8.4%	−0.01	7.5%	0.04	5.9%	0.01	9.1%	−0.03
Inflammatory arthritis	0.5%	0.01	0.6%	0.01	0.5%	0.01	0.9%	0.01
Ischemic heart disease	1.0%	0.00	1.1%	0.02	0.8%	0.02	1.7%	0.01
Hypertension	6.9%	0.01	6.6%	0.06	5.1%	0.05	5.2%	0.08
Chronic renal failure	3.1%	0.00	2.9%	0.01	2.0%	0.00	2.5%	0.02
Antiarrhythmics	3.3%	0.01	3.3%	−0.01	3.0%	0.00	3.6%	−0.03
Antidepressants	17.9%	0.00	19.2%	−0.01	19.6%	−0.04	20.5%	−0.05
Anticonvulsants	5.8%	0.01	7.0%	−0.02	5.2%	0.01	8.1%	−0.02
Oral glucocorticoids	7.1%	0.01	8.0%	−0.01	6.9%	−0.04	9.0%	−0.10
NSAIDs oral	33.5%	0.01	32.1%	0.01	6.9%	0.03	31.9%	−0.07
NSAIDs topical	17.3%	0.02	13.4%	−0.02	12.5%	0.05	20.5%	0.12
Opioids (uncombined)	11.2%	−0.01	14.2%	0.01	7.6%	−0.01	14.8%	−0.03
Opioid-nonopioid combinations	31.4%	−0.01	35.6%	−0.05	19.2%	−0.04	32.6%	−0.06
Paracetamol	44.7%	−0.01	49.8%	−0.03	28.4%	−0.02	47.0%	−0.03
Proton pump inhibitors	33.8%	0.02	35.2%	−0.02	30.5%	0.00	40.1%	−0.05
Prednisolone	6.9%	0.00	7.8%	−0.01	6.7%	−0.04	8.8%	−0.10
Statins	33.6%	0.01	34.3%	−0.01	27.6%	−0.01	37.3%	0.01
Hormone replacement therapy	2.5%	0.01	2.5%	0.01	5.2%	0.01	2.1%	−0.04
Physiotherapy	8.7%	0.05	9.7%	0.06	7.0%	0.01	13.1%	0.13

a Sample sizes stratified by IV were: knee (IV1 = 30 502, IV0 = 44 025); hip (IV1 = 7015, IV0 = 21 543); hand (IV1 = 2853, IV0 = 12 239); and shoulder (IV1 = 554, IV0 = 849).

b SMD: standardized mean difference comparing covariate prevalence (categorical variables) and means (continuous variables) across levels of instrumental variable (GP practice preference for IACI). Values closer to zero indicate better balance.

### IV analysis

**Figure 2.**
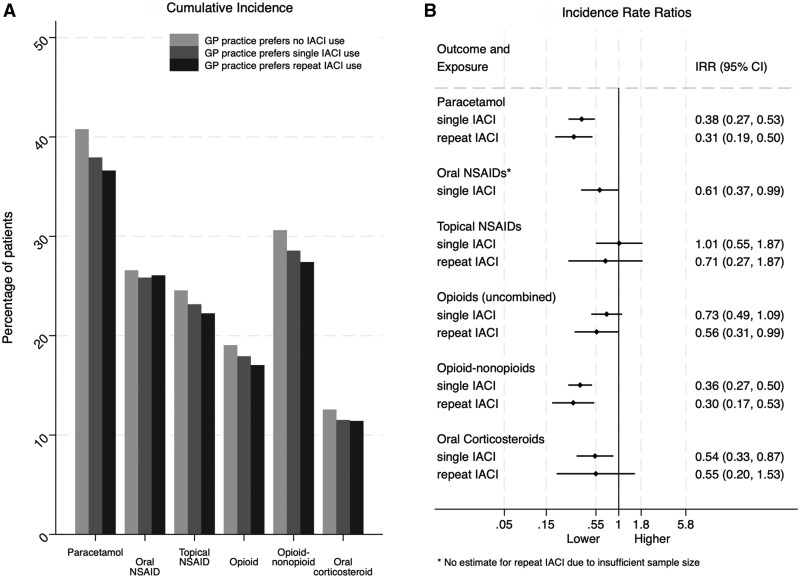
Results from instrumental variable analysis amongst knee osteoarthritis patients. (**A**) Five-year crude cumulative incidence of pain medications and (**B**) estimated effect of IACI from two-step regression model

In terms of crude associations, GP practice preference for IACI was associated with reductions in subsequent incidence of most pain medications studied amongst knee (Fig. 2A) and hand (Supplementary Fig. S1A, available at *Rheumatology* online) osteoarthritis patients. Preference for repeated IACI for knee osteoarthritis was generally associated with lower subsequent pain medication prescribing than single IACI (Fig. 2A).

Coefficients from the two-step IV regression models estimating the attributable effect of IACI use for knee osteoarthritis are shown in Fig. 2B. These indicate IACI at the knee was associated with lower incidence of most pain medications over the subsequent 5-years follow-up. Expressed as the number of patients needed to be treated to avoid the occurrence of one outcome event, single IACI for knee osteoarthritis was associated with lower incidence of opioid-nonopioid analgesic combinations [NNT = 5 (95% CI: 5–7), *P* < 0.001], oral corticosteroids [NNT = 18 (95% CI: 12–64), *P* = 0.011], paracetamol [NNT = 4 (95% CI: 3–5), *P* < 0.001] and oral NSAIDs [NNT = 10 (95% CI: 6–379), *P* = 0.046]. Slightly larger reductions were observed in several outcomes following repeated IACI for knee osteoarthritis, including: uncombined opioids [NNT = 12 (95% CI: 8–546), *P* = 0.049], opioid-nonopioid analgesic combinations [NNT = 5 (95% CI: 4–7), *P* < 0.001] and paracetamol [NNT = 4 (95% CI: 3–5), *P* < 0.001].

Coefficients from IV models estimating effect of IACI use for hand osteoarthritis are shown in Supplementary Fig. S1B, available at *Rheumatology* online. Single IACI was associated with a reduction in subsequent incidence of opioid-nonopioid analgesic combinations [NNT = 5 (95% CI: 5–9), *P* < 0.001], paracetamol [NNT = 5 (95% CI: 4–11, *P* = 0.007)] and oral NSAIDs [NNT = 5 (95% CI: 4–12), *P* = 0.008].

In sensitivity IV analyses for knee osteoarthritis, findings were similar in models adjusted for geographic region and calendar year, in models using a later index date and when using only joint-specific Read codes for identifying IACI (Supplementary Figs S2 and S3, available at *Rheumatology* online). In analyses restricted to patients aged ≥60 years who were eligible for free prescriptions, IACI was associated with larger reductions in prescribing of most pain medications (Supplementary Fig. S2C, available at *Rheumatology* online). Likewise, IACI for knee osteoarthritis was associated with large reductions in repeat (≥10) prescriptions of pain medications including uncombined opioids (Supplementary Fig. S2D, available at *Rheumatology* online). Sensitivity findings in the hand osteoarthritis cohort were identical to those in the main analysis following further adjustment and among patients aged ≥60 years (results not shown), although the sample size was inadequate for other sensitivity analyses due to model non-convergence.

### Propensity score analysis

**Figure 3.**
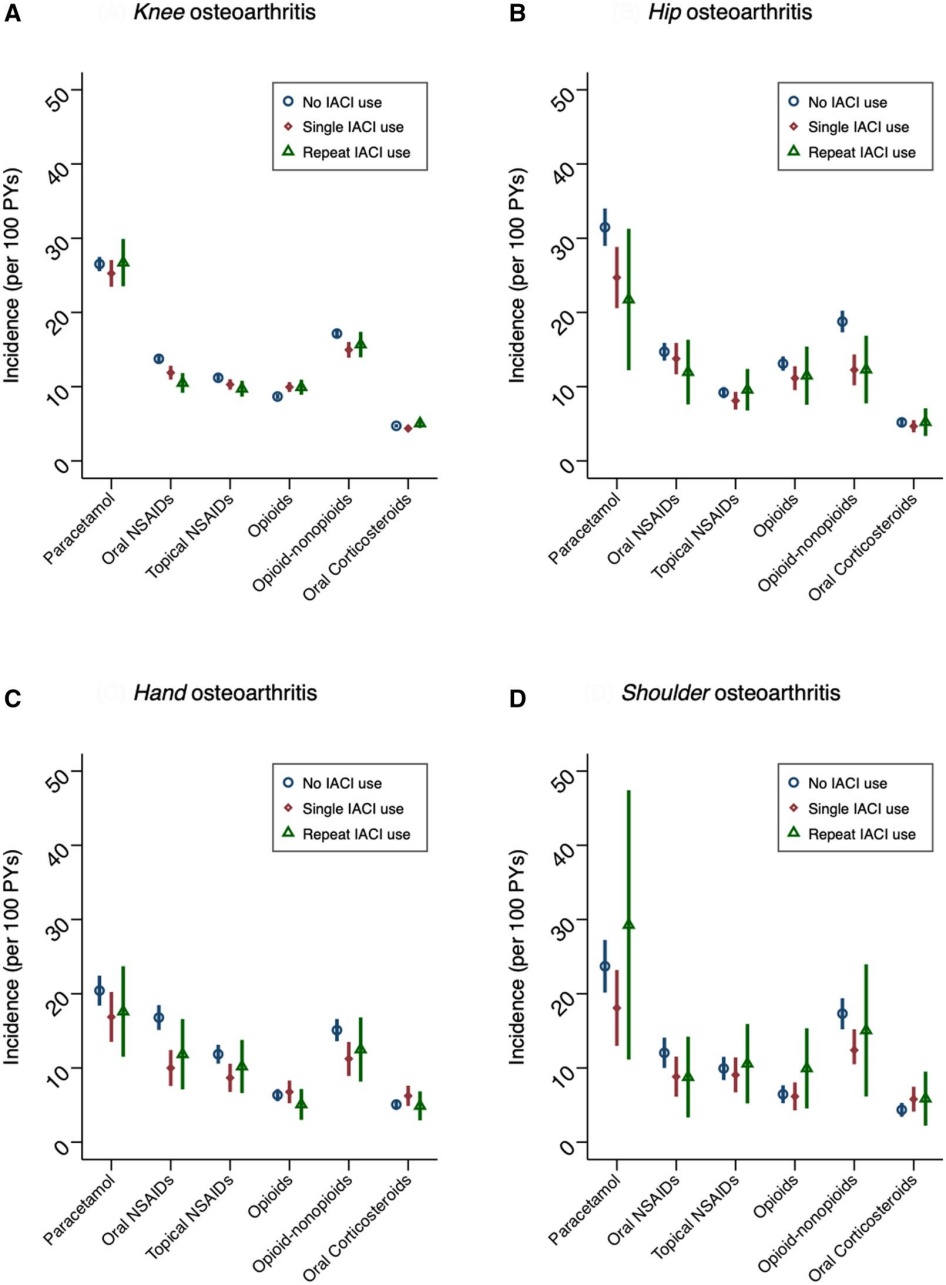
Incidence rates (95% CI) of pain medications during follow-up: results from secondary propensity score analysis

In secondary analyses accounting for measured covariates only, several estimates suggested IACI to be associated with some reduction in subsequent use of pain medicines, although most confidence intervals spanned the null and results were heterogeneous (Fig. 3). However, there was a consistent reduction in the incidence of opioid-nonopioid analgesic combinations following single IACI for knee [HR 0.88 (95% CI: 0.81, 0.96)], hip [HR 0.76 (95% CI: 0.62, 0.92)], hand [HR 0.77 (95% CI: 0.61, 0.98)] and shoulder [HR 0.72 (95% CI: 0.53, 0.99)] osteoarthritis (Supplementary Fig. S4, available at *Rheumatology* online). HRs comparing IACI exposure (single and repeated) to non-exposure for each outcome are shown in Supplementary Fig. S4, available at *Rheumatology* online.

## Discussion

### Main findings

In main analyses of single IACI for knee or hand osteoarthritis, we observed an NNT of approximately five patients to prevent one occurrence of initiating opioid-nonopioid analgesic combinations over the subsequent five years, with secondary analyses confirming reductions in opioid-nonopioid prescribing following IACI for knee, hip, hand or shoulder osteoarthritis.

### Findings in context

These findings generally support existing guidelines on the use of IACI for osteoarthritis [9, 11, 34–36]. NICE recommends use of IACI for short-term pain relief, but acknowledges evidence is limited and inconsistent [11]. Whilst existing data on IACI for hip osteoarthritis is positive [37, 38], more evidence on IACI effectiveness as non-knee joints is required and recommended [11, 15]. IACI for hand osteoarthritis has been found in RCT settings to reduce pain on movement and joint swelling at three months [39]. The European Alliance of Associations for Rheumatology (EULAR) recommendations for the management of hand osteoarthritis had initially favoured IACI use [34], although they currently advise that IACI ‘should not generally be used’ given a lack of evidence for a beneficial effect but may be considered for painful interphalangeal joints [40]. The American College of Rheumatology (ACR) guidelines conditionally recommend IACI for hand osteoarthritis [36], which is a position the European Society for Clinical and Economic aspects of Osteoporosis and Osteoarthritis (ESCEO) endorse as a patient-centric approach [41].

Worth consideration is that NICE evidence review on the topic states randomized controlled trial (RCT) data underpinning their guidance ‘does not appear to represent the diverse population of people with osteoarthritis’, and that future research should be more generalizable [11]. Furthermore, data on the relative effect of recurrent *vs* single IACI for osteoarthritis are relatively scarce [11, 13]. One high-quality RCT of knee osteoarthritis patients previously found no effect of repeated IACI on pain reduction, although pain was only measured three months after each injection and therefore any short-term effects were not detected [42]. Our findings from primary analyses on repeated IACI in knee osteoarthritis may suggest a possible cumulative benefit for recurrent (two or more injections) compared with single IACI use, but this remains a subject for further confirmation.

### Difference between primary and secondary analyses

The use of preference-based instruments is a common approach in pharmaco-epidemiology [22]. The method makes strong assumptions, but where these are upheld it provides a means of estimating treatment effect in a manner unbiased by either measured or unmeasured confounding factors, particularly useful in studies of intended effects such as this one where uncontrolled confounding would otherwise be substantial [22]. It is reassuring that we observed balance in patient covariates across IV groups for single IACI in both knee and hand osteoarthritis cohorts. However, we were unable to confirm whether the instrument was truly exogenous as this is not empirically verifiable [23]. Practice preference for IACI was also only estimated around the time of osteoarthritis diagnosis, where milder disease predicts stronger response to IACI [43].

On the other hand, there are several important caveats to the secondary propensity score matching. These models estimated the average treatment effect only in the treated population for whom there was propensity score overlap with those not receiving IACI. As such, they pertained to a much smaller and less generalizable sample. It is also probable that the patient-level matching in these models left them more prone to time-varying confounding, given patients were matched only on baseline characteristics at the date of osteoarthritis diagnosis. This matching was also not able to factor in differences in any covariates that were not available in the primary care database, therefore probably leading to residual confounding across multiple factors (as detailed in the methods section). While previous pain medication use was well balanced between matched groups, we did not assess the number of previous consultations for joint pain, which may have remained imbalanced. It is interesting to note previous studies in other settings have reported protective treatment effects that only became apparent following use of the IV approach to deal with unmeasured confounding [44].

### Possible mechanisms and implications

Overall, our findings suggest short-term pain reduction following IACI may translate to longer-term benefits in terms of less need for various pain medications after injection, including stronger medications such as opioids. There is growing awareness that greater vigilance is needed in the practice of prescribing opioids for rheumatic and musculoskeletal diseases given the potential for dependency and adverse events [45, 46]. There is also the possibility that short-term pain relief as a result of IACI could provide a ‘window of opportunity’ in which to establish therapeutic exercise and/or weight loss, which are themselves effective core treatments for osteoarthritis [9, 47].

Whilst the findings from this work should facilitate the shared decision-making process between osteoarthritis patients and clinicians when considering the use of IACIs, future confirmatory studies are needed as are those to further elucidate potential mechanisms of effect. Questions for future research include what the comparative effectiveness of IACI is to other non-surgical treatments, when in the osteoarthritis care pathway patients should be offered IACI, what the value for money of IACI is, what dose and type of IACI is most effective and what the possible impact of IACI is on the need and/or timing of surgical intervention [48].

### Strengths and limitations of the study

Although identification of osteoarthritis has been validated in CPRD GOLD, recorded date of diagnosis in relation to disease onset may not be as reliable [49]. Reliance on Read codes for identifying osteoarthritis may mean we captured a slightly later stage of disease were earlier stages recorded as consultations for joint pain without an explicit recording of osteoarthritis. While we partly addressed this in main analyses by including IACI exposure in the six months prior to diagnosis, future work is needed to investigate when in the osteoarthritis pathway patients benefit most from IACI and what dose and type of IACI is most effective. Nonetheless, CPRD GOLD provided a large and generalizable sample (on a national level) in terms of age, sex and ethnicity [16], which addresses a potential limitation of existing data from RCT settings. We did not distinguish between primary and secondary osteoarthritis, nor were we able to perform external validation of code lists used to identify IACI. We were unable to distinguish IACI administered in secondary care, which is more often performed using ultrasound or other imaging guidance. Several of the outcome medications would also have been available to buy over the counter, meaning reported associations for such medications (e.g. paracetamol) are likely to have been biased toward the null, although it is reassuring results were similar in sensitivity analyses restricted to those aged ≥60 years who were eligible for free prescriptions on the NHS. The longitudinal nature of CPRD GOLD meant longer-term outcomes could be reliably and relatively easily ascertained, compared with the RCT setting.

## Conclusions

In main analyses of people with knee or hand osteoarthritis, IACI showed lower use of most pain medications over the subsequent five years relative to no use of IACI. Secondary analyses confirmed single IACI to be associated with lower longer-term incidence of opioid-nonopioid analgesic combinations for patients with knee, hip, hand or shoulder osteoarthritis.

## Supplementary material 

Supplementary material is available at *Rheumatology* online.

## Supplementary Material

keaf126_Supplementary_Data

## Data Availability

The data underlying this submission were provided by CPRD under licence/under permission. Applications to access CPRD GOLD and linked data must be made directly to CPRD in accordance with CPRD’s RDG process.
