# Supplementary material for: Effect of intra-articular corticosteroid injections for osteoarthritis on the subsequent use of pain medications: a UK CPRD cohort study
Source: Rheumatology (Oxford). 2025 Mar 1;64(6):3832–41. doi: 10.1093/rheumatology/keaf126 (PMC12107031; doi:10.1093/rheumatology/keaf126)

Supplementary Items for: *'Effect of intra-articular corticosteroid injections for osteoarthritis on the subsequent use of pain medications: a UK CPRD cohort study'*

Authors: Samuel Hawley, Albert Prats-Uribe, Gulraj S Matharu, Antonella Delmestri, Daniel Prieto-Alhambra, Andrew Judge, Michael R Whitehouse

CONTENTS

Supplementary Table 1: Population flow for instrumental Variable (IV) analysis

Supplementary Table 2: Population flow for propensity score matched sensitivity analysis

Supplementary Figure 1: (A) 5-year cumulative incidence of pain medications following *hand* osteoarthritis diagnosis and (B) estimated effect of *hand* IACI using two-step regression

Supplementary Figure 2: Estimated effect of IACI from supplementary analysis IV models amongst *knee* osteoarthritis patients: (A) adjusted for geographic region and calendar year, (B) using model index date of 1-year after OA diagnosis data, (C) only including patients aged 60 years or over, and (D) using an outcome of repeat rather than incident pain prescriptions

Supplementary Figure 3: Estimated effect of IACI from supplementary analysis IV model amongst *knee* osteoarthritis patients: defining IACI exposure using only knee-specific injection codes (i.e. not using injection codes lacking joint site of administration)

Supplementary Figure 4: Estimated effect of IACI use on subsequent initiation of pain medication: results from propensity score (PS) matched secondary analyses (pooled across multiple imputed datasets)

Supplementary Table 1: Population flow for instrumental variable (IV) analysis

	KNEE		HIP		HAND		SHOULDER		TOTAL	
Exclusion criteria/reason	Sample size	Count	Sample size	Count	Sample size	Count	Sample size	Count	Sample size	Count
	120,596		60,966		33,542		7758		222,862	
Aged <20 years		54		31		9		0		94
BMI <15		14		18		11		5		48
Orthopaedic surgery prior to OA date		4823		2508		1027		414		8772
OA date after 31/12/2019		1284		908		402		116		2710
Osteoarthritis at multiple joints		13,706		9996		5722		2268		31692
Censored (died or lost to follow-up) prior to index date		4222		2079		1062		260		7623
Replacement, arthroscopy or debridement prior to index date		3230		2639		-		-		5869
Steroid injection prior to 1 year before index date		3834		969		637		490		5930
	90,924		42,843		25,027		4447		163,241	
Additional exclusions for single IACI cohort only										
repeat injection received		1,042		73		74		39		1228
IV 'burn in' period		15,355		14,212		9861		3005		42433
	74,527		28,558		15,092		1403		119,580	
Prevalent use of outcome prescription in each respective analysis		various		n/a		various		n/a		various
Oral NSAIDs analysis	40,528		n/a		9,792		n/a		50,320	
Opioids (uncombined) analysis	62,351		n/a		13,607		n/a		75,958	
Oral Corticosteroids analysis	67,675		n/a		13,670		n/a		81,345	
Paracetamol analysis	30,804		n/a		9,414		n/a		40,218	
Opioid-nonopioids analysis	42,858		n/a		11,212		n/a		54,070	
Topical NSAIDs analysis	54,592		n/a		10,416		n/a		65,008	
*Additional exclusions for repeat IACI cohort only**										
single IACI received		4,080		738		411		278		5507
IV 'burn in' period		15,307		14,155		9,838		2902		42202
	71,537		27,950		14,778		1267		115,532	
Prevalent use of outcome prescription in each respective analysis		various		n/a		n/a		n/a		various
Oral NSAIDs analysis	39,001		n/a		n/a		n/a		39,001	
Opioids (uncombined) analysis	60,068		n/a		n/a		n/a		60,068	
Oral Corticosteroids analysis	71,533		n/a		n/a		n/a		71,533	
Paracetamol analysis	71,526		n/a		n/a		n/a		71,526	
Opioid-nonopioids analysis	41,346		n/a		n/a		n/a		41,346	
Topical NSAIDs analysis	52,507		n/a		n/a		n/a		52,507	

<u>Supplementary Table 2: Population flow for propensity score matched secondary analysis</u>

Exclusion criteria/reason	KNEE		HIP		HAND		SHOULDER		TOTAL	
	Sample size	Count	Sample size	Count	Sample size	Count	Sample size	Count	Sample size	Count
	120,596		60,966		33,542		7758		222,862	
Aged <20 years		54		31		9		0		94
BMI <15		14		18		11		5		48
Orthopaedic surgery prior to OA date		4823		2508		1027		414		8772
OA date after 31/12/2019		1284		908		402		116		2710
osteoarthritis at multiple joints		13,706		9996		5722		2268		31692
Steroid injection prior to index date		4990		1,213		723		612		7538
	95,735		46,705		26,700		5166		174,306	
Not matched		various		various		various		various		various
Oral NSAIDs analysis*	15,869		3345		2004		901		22,119	
Opioids (uncombined) analysis*	20,688		4185		2448		1117		28,438	
Oral Corticosteroids analysis*	22,358		4842		2625		1221		31,046	
Paracetamol analysis*	12,666		2445		1848		712		17,671	
Opioid-nonopioids analysis*	16,237		3210		2121		773		22,341	
Topical NSAIDs analysis*	19,826		4671		2379		1149		28,025	

* Figures pertain to one full imputation

Supplementary Figure 1: (A) 5-year cumulative incidence of pain medications following *hand* osteoarthritis diagnosis and (B) estimated effect of *hand* IACI using two-step regression

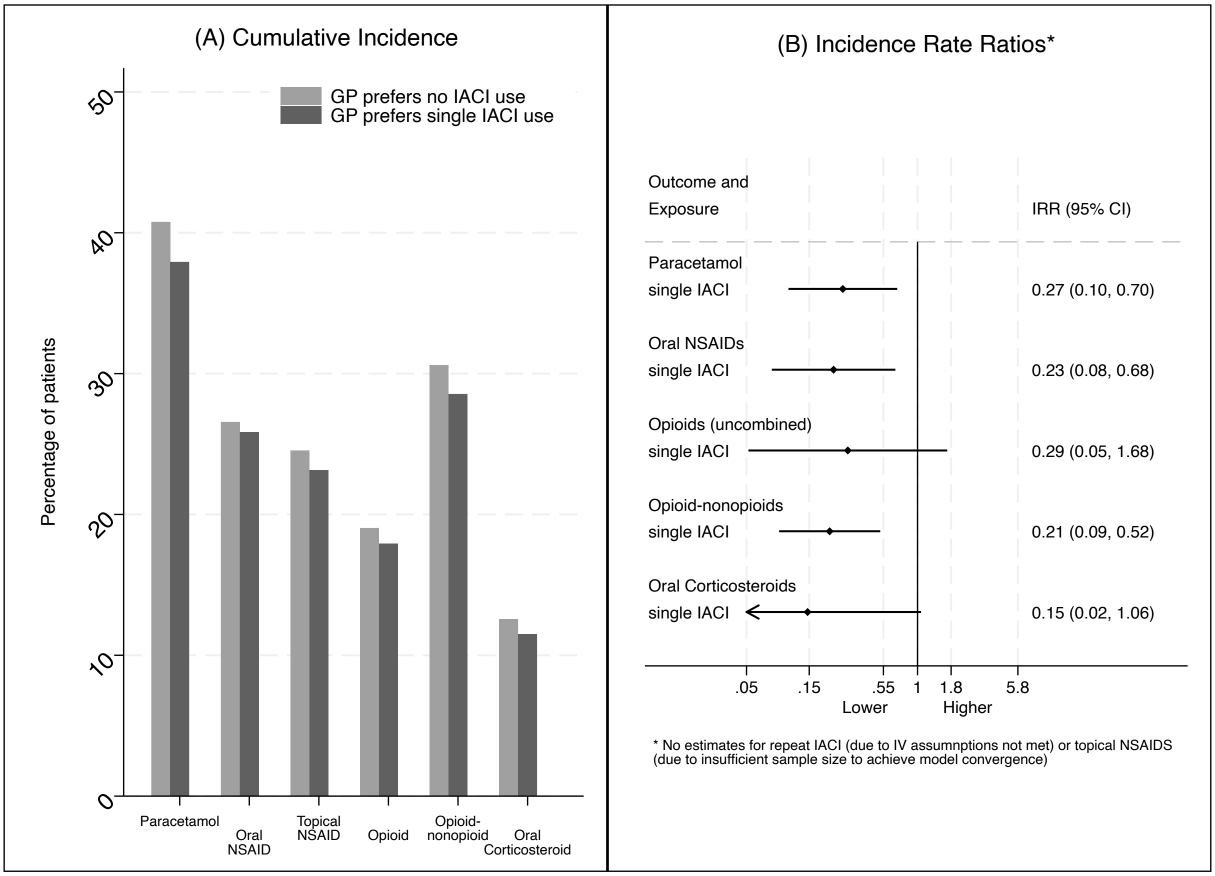

Supplementary Figure 2: Estimated effect of IACI from supplementary analysis IV models amongst *knee* osteoarthritis patients: (A) adjusted for geographic region and calendar year, (B) using model index date of 1-year after OA diagnosis data, (C) only including patients aged 60 years or over, and (D) using an outcome of repeat rather than incident pain prescriptions

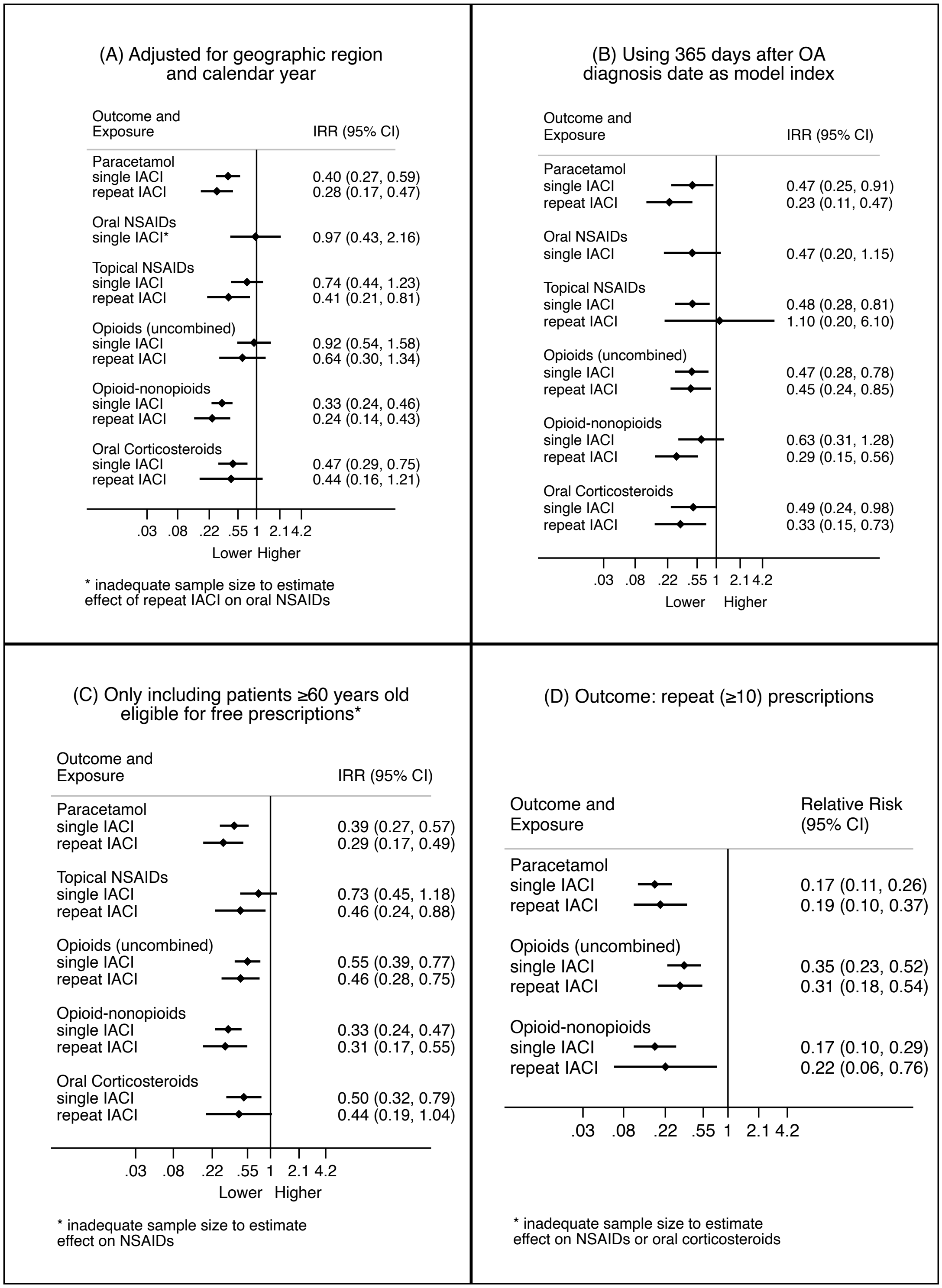

Supplementary Figure 3: Estimated effect of IACI from supplementary analysis IV model amongst *knee* osteoarthritis patients: defining IACI exposure using only knee-specific injection codes (i.e. not using injection codes lacking joint site of administration)

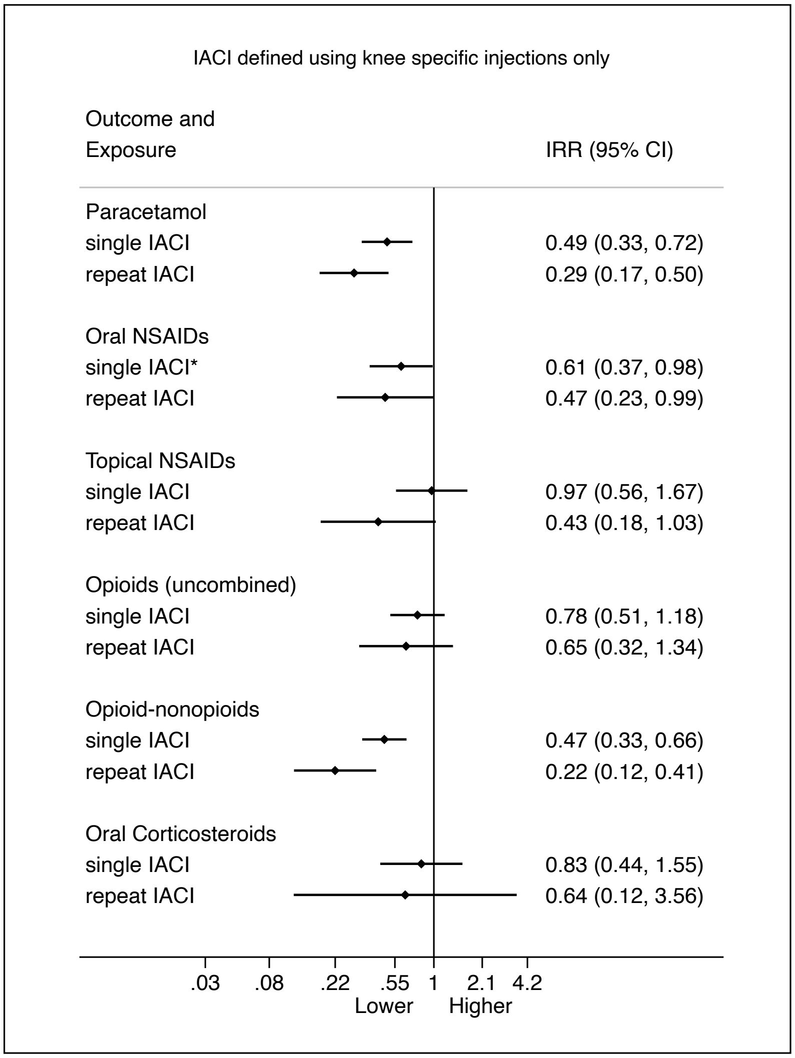

Supplementary Figure 4: Estimated effect of IACI use on subsequent initiation of pain medication: results from propensity score (PS) matched secondary analyses (pooled across multiple imputed datasets

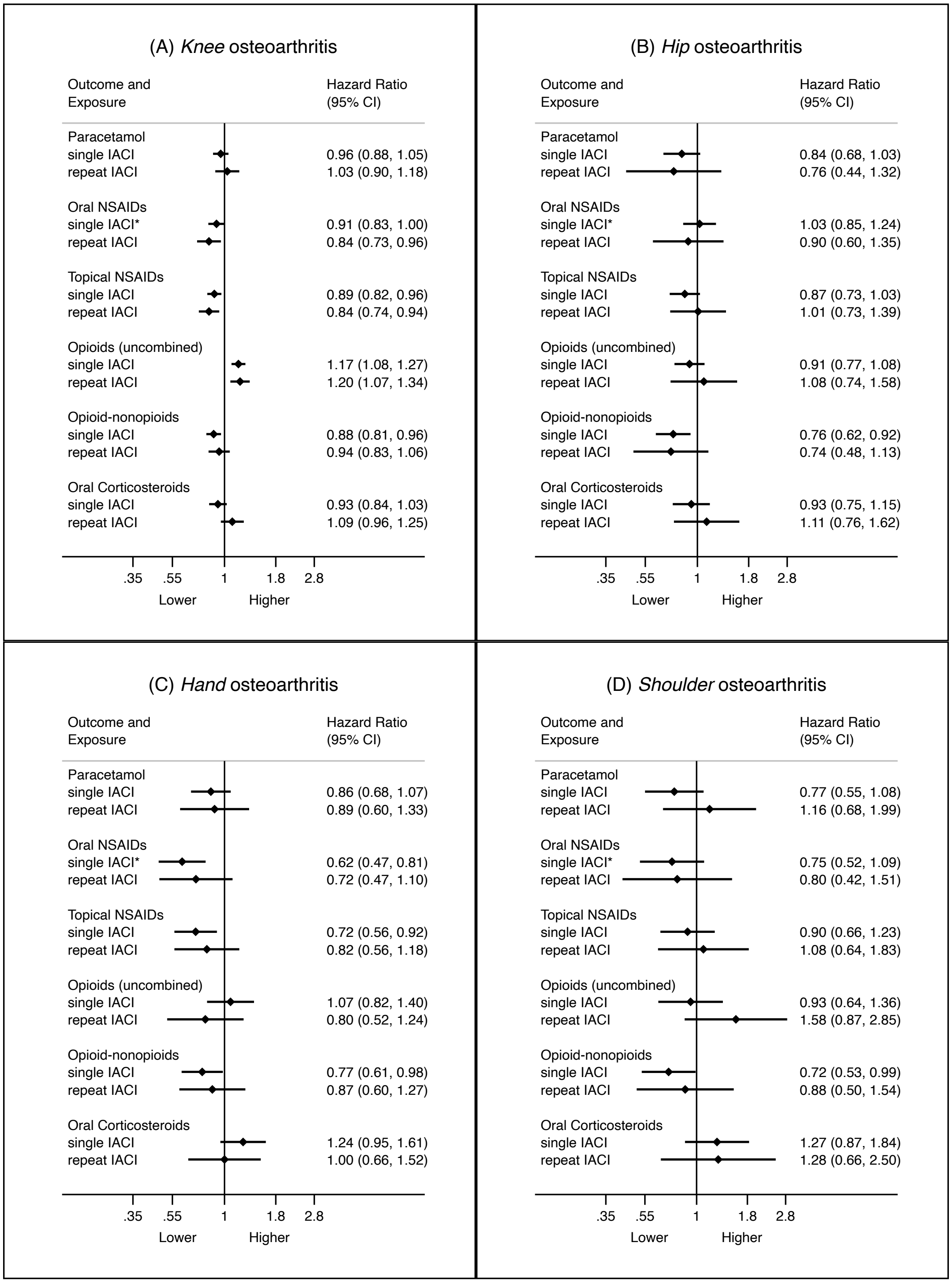

Supplement: keaf126_Supplementary_Data [file keaf126_supplementary_data.pdf]
